# Protein Kinase A-Dependent Plasticity of Local Inhibitory Synapses from Hilar Somatostatin-Expressing Neurons

**DOI:** 10.1523/ENEURO.0089-23.2023

**Published:** 2023-10-03

**Authors:** Alicia Hernández-Vivanco, Esther Jiménez-Redondo, Nuria Cano-Adamuz, Pablo Méndez

**Affiliations:** Instituto Cajal, Consejo Superior de Investigaciones Científicas (CSIC), 28002 Madrid, Spain

**Keywords:** hippocampus, inhibitory neuron, parvalbumin, somatostatin, synaptic inhibition, synaptic plasticity

## Abstract

Hippocampal inhibitory neurons (INs) contact local targets and project to different brain areas to form synapses on distal neurons. Despite the importance of INs for hippocampal function and interregional brain communication, the impact of activity-dependent plasticity mechanisms on local and long-range GABAergic synapses formed by hippocampal INs remains to be fully elucidated. Here, we use optogenetic-coupled electrophysiology in mice to show that protein kinase A (PKA), a master regulator of GABAergic synapse plasticity, causes a form of long-term potentiation of inhibitory synapses (iLTP) in hippocampal granule cells (GCs). This form of iLTP is observed in GCs synapses originated in local INs expressing the marker somatostatin (SST), but not in those expressing parvalbumin. Long-range synapses formed by SST INs onto medial septum neurons are unaffected by PKA activation. iLTP of local SST synapses on GCs is accompanied by changes in presynaptic probability of release and is occluded by pharmacological increase of synaptic activity *in vivo*. Our results suggest that PKA-dependent inhibitory synapse plasticity is expressed in local, but not long-range, targets of SST INs and selectively modifies inhibitory microcircuits essential for hippocampal function.

## Significance Statement

The hippocampus, a brain region essential for memory, is populated by a diverse group of inhibitory neurons (INs) that form synaptic contacts onto local principal cells and long-range targets. Changes in inhibitory synapse strength shape local and interregional brain communication. However, how synapse plasticity mechanisms are implemented in the rich assortment of hippocampal INs remains to be fully defined. Here we show that protein kinase A, a signaling protein involved in memory, enhances inhibitory neurotransmission from local, but not long-range, projecting hippocampal INs expressing the molecular marker somatostatin. The consequences of this plasticity are observed after increasing neuronal activity *in vivo*. Our work describes a form of activity-dependent synapse plasticity that regulates inhibitory microcircuits essential for hippocampal memory function.

## Introduction

Inhibitory synapses in hippocampal excitatory neurons originate in a diverse array of presynaptic inhibitory neurons (INs) subtypes with specific roles in network activity, spatial navigation, and memory (Klausberger and Somogyi, 2008). As their excitatory counterparts, inhibitory synapses, which use GABA as a neurotransmitter, undergo several forms of plasticity that modify their structure and function (Castillo et al., 2011; Kullmann et al., 2012; Flores and Méndez, 2014). Inhibitory synapses are endowed with a wide variety of plasticity mechanisms that parallels their molecular and functional heterogeneity (Méndez and Bacci, 2011). Defining the mechanisms of hippocampal GABAergic synapse plasticity in the context of IN subtype assortment is essential to refine our basic understanding of IN diversity and the role of inhibitory synapse plasticity in learning and memory.

In the hippocampal dentate gyrus (DG), INs expressing the molecular markers somatostatin (SST) and parvalbumin (PV) provide a large fraction of synaptic inhibition to local glutamatergic granule cells (GCs; Houser, 2007; Hosp et al., 2014). Although they share common molecular features and developmental origin (Lim et al., 2018), SST and PV INs are heterogenous classes and comprise several subtypes with different target specificities at the level of subcellular compartment and target regions (Harris et al., 2018). Dentate SST INs form synaptic contacts onto local GC dendrites (Hosp et al., 2014) and send long-range axon collaterals to the medial septum (MS; Jinno, 2009). Different types of PV INs provide perisomatic inhibition by impinging on the soma, proximal dendrites, and axon initial segments of target neurons (Hu et al., 2014). This synapse-specific features have been proposed to determine the functional impact of inhibitory synapse plasticity on principal excitatory neurons o9(Méndez and Bacci, 2011).

Protein kinase A (PKA) is a master regulator of synaptic plasticity of GABAergic synapses across multiple brain regions (Castillo et al., 2011). PKA regulates postsynaptic receptors acting on inhibitory synapse scaffolding proteins and receptors (Poisbeau et al., 1999; Nugent et al., 2009). In addition, PKA has presynaptic mechanisms of action, including the regulation of neurotransmitter release (Monday et al., 2018). The activity of PKA is tightly linked to neuronal activity through different neuromodulators, glutamate receptors, and intracellular calcium levels (Greengard, 2001; Halls and Cooper, 2011).

Here we tested the existence of IN subtype-specific mechanisms of PKA-dependent GABAergic synapse plasticity. For this, we performed electrophysiological recordings of GABAergic synapses formed by two major types of DG INs (PV and SST INs) in local (i.e., GCs) and long-range (i.e., medial septum) target neurons. We found that PKA activity induced by pharmacological rise of cAMP levels triggers a form of long-term potentiation that affects local but not long-range synapses formed by SST DG INs. In addition, we tested the dependency of this form of plasticity on previous increases in neuronal activity using *in vivo* treatment with kainic acid (KA). Our results suggest that the induction of PKA-dependent plasticity of GABAergic synapses interacts with neuronal activity and could be part of experience-dependent modifications of DG inhibitory circuits.

## Materials and Methods

### Animals

Group-housed adult male and female C57BL/6J wild-type, SST-Cre [Ssttm2.1(cre)Zjh/J], and PV-Cre [Pvalb tm1(cre)Arbr/J] mice maintained in a 12 h light/dark cycle with unlimited access to food and water were used for the study (45% males, 55% females). Mice were killed at 8–12 weeks for electrophysiological recordings. All procedures were performed according to protocols approved by the Institutional Animal Care and Use Committee of the Cajal Institute and approved by the local veterinary office (Comunidad de Madrid).

### Reagents and adeno-associated viruses

Forskolin (FSK) and PKI14-22 (both from Tocris Bioscience) were dissolved in DMSO (FSK, 10 mm; PKI14-22, 1 mm). H89 (Tocris Bioscience) was dissolved in distilled water. Previous to use, the compounds were further dissolved in artificial CSF (ACSF) to final concentration (FSK, H89: 10 μm; PKI14-22: 1 μm). Kynurenic acid (Sigma-Aldrich) and SR-95531 (gabazine, Tocris Bioscience) were dissolved in ACSF at final concentrations of 2 mm and 10 μm, respectively. KA (Sigma-Aldrich) was dissolved in saline solution to 2 mg/ml and used at a final dose of 5 mg/kg. Adeno-associated viruses (AAVs) were used with serotype 5 and purchased from the University of North Carolina Vector Core (AAV-EF1a-DIO-ChETA-EYFP).

### Surgery

Analgesic (paracetamol 0.2 g/kg) treatment was administered for 4 d around surgery. Under anesthesia (isoflurane: induction, 5%; maintenance, 1.5–2.0%), mice were placed in a stereotaxic frame (RWD), and one craniotomy (left hemisphere) was performed using the following stereotaxic coordinates adopted to target the dorsal DG: anterior–posterior, −2.2; medial–lateral, −1.45; dorsal–ventral, −1.9. Injections of AAV (0.5–0.6 μl) were performed using graduated pipettes (Drummond Scientific Company), pulled and broken back to a tip diameter of 10–15 μm, at an infusion rate of ∼0.05 μl/min. Micropipettes were left in place for 5 min following microinjection and slowly retracted (0.4 mm/min) to avoid reflux of the viral solution. Experiments involving AAVs started on the third week after the viral injection.

### Kainate treatment

Mice were treated with two to three intraperitoneal injections of kainate at low dose (5 mg/kg) at 30 min intervals. The behavior of the animals was monitored between injections and was rated according to Racine (1972) until behavior compatible with stages 2 and 3 (head nodding and forelimb clonus) was observed. Mice were processed for inmunohistochemistry or electrophysiological recordings 90 min after the last KA injection.

### Electrophysiology

**Figure 1.**
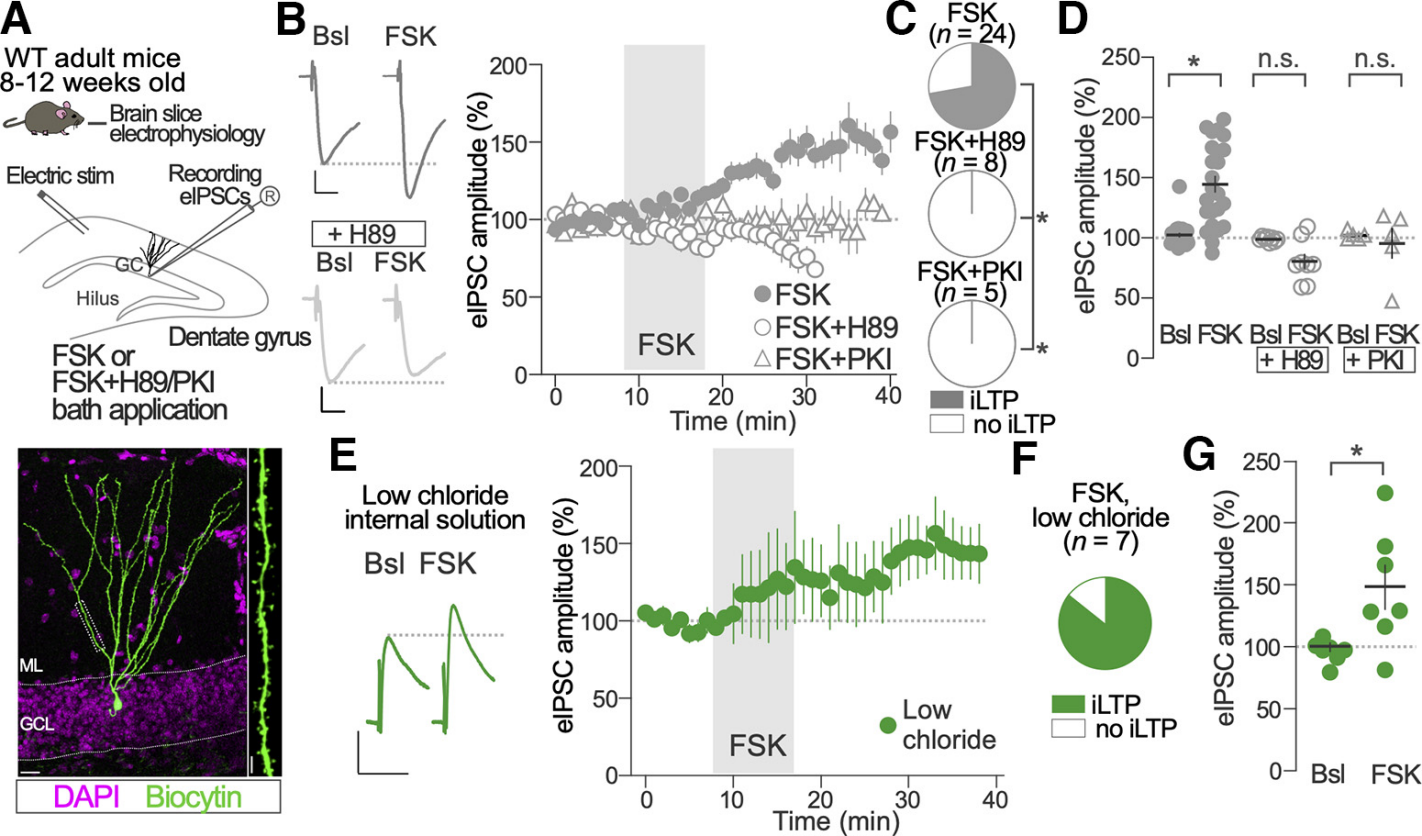
Protein kinase A activation induces iLTP on hippocampal GCs. ***A***, Top, Schematic of experimental procedure. Bottom, Representative example of a biocytin-filled GC (green) recorded during the experiments. The boxed region is magnified in the right part of the panel and shows a dendritic spine covered dendritic segment. Scale bars: bottom panel, left, 20 μm; bottom panel, right, 5 μm. ***B***, Representative traces of electrically eIPSCs before (Bsl) and after FSK (10 μm) bath application in the absence or presence of PKA inhibitor H89 (+H89, 10 μm). Calibration: 50 ms, 50 pA. The time course of FSK effects on normalized eIPSC amplitude in the absence (FSK) or presence of PKA inhibitors H89 (+H89, 10 μm) and PKI14-22 (+PKI, 1 μm) is shown in the right graph. ***C***, Parts of whole graphs represent the proportion of recorded GCs showing significant increase of eIPSC amplitude after FSK application in the absence (FSK) or presence of PKA inhibitors H89 (+H89, 10 μm) and PKI14-22 (+PKI, 1 μm). Two-sided Fisher’s exact test; FSK versus FSK+H89, *p* = 0.002; FSK versus FSK+PKI, *p* = 0.002; FSK, *n* = 24 GCs; FSK+H89, *n* = 8 GCs; and FSK + PKI, *n* = 5 GCs. ***D***, Population data of FSK effects on normalized eIPSC amplitude before (Bsl) and 20 after FSK application in the presence and absence of PKA inhibitors H89 and PKI14-22. Two-way ANOVA; Treatment (FSK, FSK+H89, FSK+PKI): *F*_(2,34)_ = 13.86, *p* = 0.0001; Bonferroni’s multiple-comparisons test; Bsl versus FSK, *p* < 0.0001; Bsl versus FSK+H89, *p* = 0.27; Bsl versus FSK+PKI, *p* > 0.99; FSK, *n* = 24 GCs; FSK+H89, *n* = 8 GCs; FSK+PKI, *n* = 5 GCs. ***E***, Representative traces of eIPSC before (Bsl) and after FSK (10 μm) bath application recorded with a low-chloride internal solution. The time course of FSK effects is shown in the right graph. ***F***, Proportion of GCs recorded with low chloride showing a significant increase of eIPSC amplitude after FSK application. ***G***, Population data of FSK effects on normalized eIPSC amplitude before (Bsl) and after FSK application in the low-chloride condition. Two-sided *t* test; *t*_(12)_ = 2.772, *p* = 0.02; *n* = 7 GCs. *p < 0.05; n.s., nonsignificant. Dots and vertical bars in ***B*** and ***E***, and horizontal and vertical bars in ***D*** and ***G*** represent the mean ± SEM. Dots in ***D*** and ***G*** represent individual measurements.

**Figure 2.**
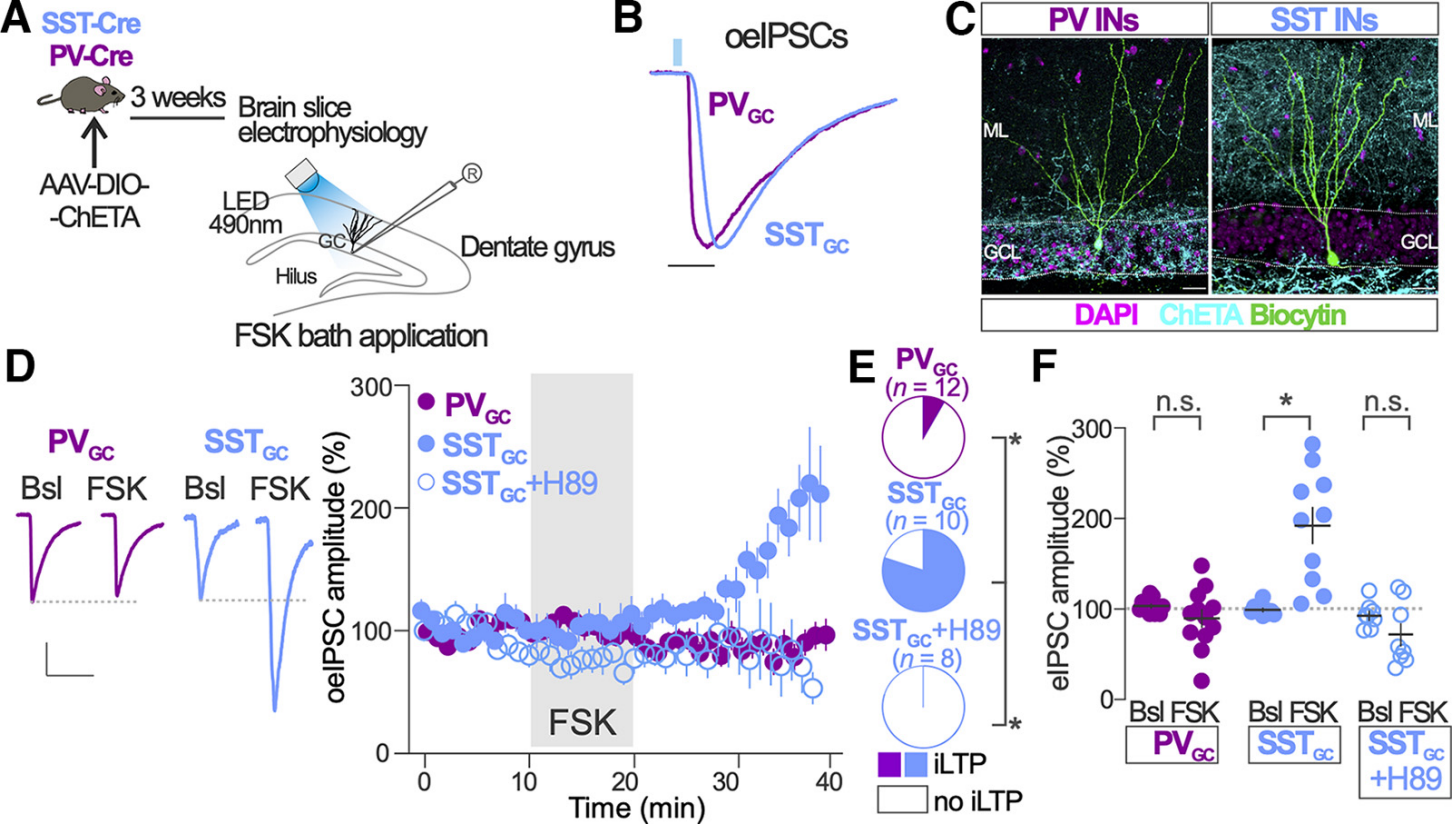
PKA-induced iLTP is observed in GABA synapses originated in SST but not in PV INs. ***A***, Schematic of experimental procedure. ***B***, Representative traces of normalized oeIPSCs recorded in GCs from PV-Cre and SST-Cre mice infected with AAV-DIO-ChETA-EYFP. The blue light (490 nm) pulse used to elicit oeIPSCs is represented with a vertical blue bar. Scale bar, 10 ms. ***C***, Representative images of Cre-dependent ChETA expression (cyan) and biocytin-filled GC (green) in the DG of slices prepared from PV-Cre and SST-Cre mice. Scale bar, 25 μm. ***D***, Representative traces of oeIPSCs before (Bsl) and after FSK bath application in GCs from PV-Cre (PV_GC_) and SST-Cre (SST_GC_) mice. Calibration: 50 ms; 30 pA (SST-Cre) and 50 pA (PV-Cre). The time course of FSK effects on normalized oeIPSC amplitude in both conditions is shown in the right graph. Open symbols correspond to FSK application in the presence of PKA inhibitor H89 in experiments performed in SST-Cre mice (SST_GC_+H89). ***E***, Parts of whole graphs represent the proportion of recorded GCs showing significant increase of eIPSC amplitude after FSK application in PV-Cre and SST-Cre mice in the absence and presence of PKA inhibitor H89. Two-sided Fisher’s exact test; SST_GC_ versus PV_GC_, *p* = 0.002; SST_GC_ versus SST_GC_+H89, *p* = 0.0007; PV-Cre, *n* = 12 GCs; SST-Cre, *n* = 10 GCs; SST-Cre + H89, *n* = 8 GCs. ***F***, Population data of FSK effects on normalized oeIPSC amplitude before (Bsl) and after FSK application in GCs from PV-Cre and SST-Cre mice. Two-way ANOVA; treatment/genotype: *F*_(2,27)_ = 20.64, *p* < 0.0001; Bonferroni’s multiple-comparisons test; PV-Cre Bsl versus FSK, *p* = 0.99; SST-Cre Bsl versus FSK, *p* < 0.0001; SST-Cre+H89 Bsl versus FSK, *p* = 0.71; PV-Cre mice, *n* = 12 GCs; SST-Cre mice, *n* = 10; SST-Cre+H89, *n* = 8 GCs. *p < 0.05; n.s., nonsignificant. Dots and vertical bars in ***D***, and horizontal and vertical bars in ***F*** represent the mean ± SEM. Dots in ***F*** represent individual measurements.

Acute slices for electrophysiological recordings were prepared from 8- to 10-week-old male and female mice. Brains were quickly removed, and coronal slices containing the dorsal hippocampus (300 μm) or the medial septum (250 μm) were cut at 4°C with a vibratome in a solution containing the following: 234 mm sucrose, 11 mm glucose, 26 mm NaHCO_3_, 2.5 mm KCl, 1.25 mm NaH_2_PO_4_, 10 mm MgSO_4_, and mM 0.5 CaCl_2_ (equilibrated with 95% O_2_ and 5% CO_2_). Recordings were obtained at 30–32°C from DG GCs visually identified using infrared video microscopy. Recordings were performed in ACSF equilibrated with 95% O_2_ and 5% CO_2_ containing 126 mm NaCl, 26 mm NaHCO3, 2.5 mm KCl, 1.25 mm NaH_2_PO_4_, 2 mm MgSO_4_, 2 mm CaCl_2_, and 10 mm glucose, pH 7.4. To isolate GABAergic currents resulting from electrical and optogenetic stimuli, the wide-spectrum glutamate receptor blocker kynurenic acid (2 mm) was added to the recording solution in all experiments. Patch-clamp electrodes contained a “high-chloride” intracellular solution composed of the following: 60 mm Cs methane sulfonate, 70 mm CsCl, 10 mm HEPES, 5 mm EGTA, 4 mm QX-314, and 4 mm Mg ATP, pH 7.3, corrected with CsOH (290 mOsm). In a set of experiments (Fig. 1*E*,*F*), we used a “low-chloride” solution composed of 127 mm Cs methane sulfonate, 2 mm CsCl, 10 mm HEPES, 5 mm EGTA, 4 mm QX-314, and 4 mm Mg ATP, pH 7.3, corrected with CsOH (290 mOsm). The reversal potential for chloride (E_Cl_) is ∼16 mV in high-chloride solutions and −80 mV in low-chloride solutions based on the Nernst equation, without correction for liquid junction potential. All recordings were performed in the voltage-clamp configuration at a holding potential of –70 mV, resulting in inward and outward responses to GABA_A_ receptor activation in high-chloride and low-chloride conditions, respectively. Biocytin (2 mg/ml; Sigma-Aldrich) was added to a set of experiments to recover the morphology of the recorded neurons [Figs. 1*A*, 2*C* (see also Fig. 4*C*)]. All drugs were dissolved to final concentration in ACSF and applied through a gravity-driven bath perfusion system at flow rate of ∼2–3 ml/min. Electrically evoked postsynaptic currents were elicited with a bipolar theta-glass pipette filled with ACSF positioned in the outer portion of the molecular layer (ML). For optogenetic stimulation, we used a, LED source (pE-300white, CoolLED) filtered at 490 nm and directed to the channelrhodopsin-2-expressing neurons through the objective of the microscope. The average LED power (percentage of full LED light source) was 38% in hippocampal slices from SST-Cre mice, 46% in hippocampal slices from PV-Cre mice, and 2% in medial septum slices from SST-Cre mice. Signals were amplified using a Multiclamp 700B Patch-Clamp Amplifier (Molecular Devices), sampled at 20 kHz, filtered at 10 kHz, and stored on a PC. Data were analyzed using pClamp software (Molecular Devices). For illustration purposes, we used the average of five consecutive sweeps.

### Immunohistochemistry and imaging

Mice were injected with a lethal dose of pentobarbital (150 mg/kg) and underwent transcardiac perfusion with cold PBS and 4% paraformaldehyde (PFA) solution. Brains were extracted and submerged in fixative for 2 h at 4°C. Coronal 50-μm-thick sections were blocked in PBS with 10% BSA and 0.3% Triton X-100 followed by overnight incubation in with the primary antibody raised against c-Fos (1:5000; Synaptic Systems). After 3× 15 min wash in PBS at room temperature, brain sections were incubated in PBS and 0.3% Triton X-100 with Alexa-Fluor 488-conjugated anti-rabbit secondary antibody (1:500; Abcam). After three more steps of washing in PBS, the samples were mounted and covered on microscope slides using DAPI-containing mounting medium.

Slices used for electrophysiological experiments containing biocytin-loaded neurons were fixed in 4% PFA for 24 h at 4°C, blocked as described above, and incubated overnight with Alexa Fluor 633-Streptavidin (1:500; Abcam). After washing, slices were incubated with DAPI and mounted using mounting medium. Images were obtained with a confocal microscope (model SP5, Leica; with LAS AF software, Leica) using 10× or 20× objectives and 405, 488, 561, and 633 nm laser excitation wavelengths. Images (1024 × 1024 pixel, resolution 1.3 – 2.6 pixel/μm were collected with a 3 – 4 μm step size. Microcopy images in Figures 1 and 2 (and see Fig. 5)] were obtained by maximal intensity projections of three to six optical sections.

### Statistical analysis

Values are given as the mean ± SEM. In addition, we show individual measurements in each experiment. We used recorded neurons as “*n*” in electrophysiological experiments. Criteria for significant increase of electrically evoked IPSC (eIPSC) or optogenetically eIPSC (oeIPSCs) amplitude was stablished as a *z* score >2 SDs of 8–10 min baseline (Bsl) recorded normalized eIPSCs or oeIPSCs. Values of IPSC amplitude and paired-pulse ratio (PPR) used for statistical analysis were obtained averaging the 5 last min of baseline and post-treatment condition in each experiment. Standard two-tailed *t* tests were performed to compare normally distributed data points; otherwise, we used the nonparametric Mann–Whitney test. Two-way ANOVA followed by Bonferroni’s *post hoc* comparisons were used when noted. Fisher’s exact test was used to compare proportions. For all tests, we adopted an α level of 0.05 to assess statistical significance. Statistical analysis was performed using Prism (GraphPad Software). Experimenters were not blind to condition during data acquisition and analysis.

## Results

### PKA activation induces long-term potentiation of inhibitory synapses (inhibitory long-term potentiation) on hippocampal GCs

cAMP and PKA regulate synaptic plasticity in multiple brain regions. However, their impact on plasticity of GABAergic synapses in hippocampal DG GCs remains unresolved. To address this question, we pharmacologically manipulated cAMP levels and PKA activity during whole-cell voltage-clamp recordings of GABAergic synapse activity in GCs in acutely prepared slices from young adult male and female mice (Fig. 1*A*). We recorded GABA_A_ receptor-mediated transmission using a high-chloride internal solution in the presence of a glutamatergic receptor blocker (kynurenic acid, 2 mm). The addition of biocytin to the internal solution allowed the morphologic reconstruction of the recorded GCs. Reconstructed GCs had well developed dendritic arborization with numerous ramifications and dendrites covered with dendritic spines (Fig. 1*A*), suggesting that recordings were performed from mature GCs.

GABA_A_ receptor-mediated eIPSCs were evoked every 30 s using a bipolar electric stimulation electrode placed in the outer third part of the ML (Fig. 1*A*). After 8–10 min baseline period of stable amplitude eIPSCs recordings, the adenylate cyclase activator FSK (10 μm) was applied for 10 min through the perfusion bath and recordings were maintained for 20 additional minutes. Transient application of FSK induced an increase in the amplitude of eIPSCs (Fig. 1*B*, FSK) in 18 of 24 recorded GCs (Fig. 1*C*, FSK). The normalized eIPSC amplitude changed from 102 ± 2% (Bsl) to 144 ± 7% after FSK application (*p *<* *0.0001; Fig. 1*D*). To test the dependency of FSK-induced effects on PKA activity, we performed recordings in the presence of the PKA inhibitors H89 (10 μm) and myristoylated PKI14-22. In these conditions, FSK failed to alter eIPSC amplitude (H89: Bsl 99 ± 1%; FSK 80 ± 6%; *p *=* *0.27; PKI14-22: Bsl, 102 ± 2%; FSK, 95 ± 13%; *p *>* *0.99) in all recorded GCs (H89, *n* = 8; PKI14-22, *n* = 5; Fig. 1*C*,*D*). To test the potential effect of PKA inhibition on basal synaptic activity, we recorded eIPSCS after transient application of H89 (10 μm, 10 min). We failed to detect a significant effect of H89 in the amplitude of eIPSCs in GCs (Bsl, 99 ± 2%; H89, 91 ± 4%; Mann–Whitney test: *U *=* *6, *p *=* *0.22; *n *=* *5).

We performed additional recordings using an internal solution with low-chloride concentration imposing a physiological driving force for chloride currents (see Materials and Methods). In this condition, FSK application induced inhibitory long-term potentiation (iLTP) of comparable magnitude (Bsl, 96 ± 3%; FSK, 146 ± 17%; *p *=* *0.02) in a similar proportion of GCs tested (six of the seven recorded GCs; Fig. 1*E–G*) compared with the high-chloride condition.

Altogether, these results suggest that increasing cAMP production induces a form of iLTP on GABAergic synapses of GCs through PKA activation. This form of iLTP is observed at physiological chloride concentration.

### PKA-induced iLTP is observed in GABA synapses originated in SST but not in PV INs

GABAergic synapses in GCs originate from two major IN types: the SST- and the PV-expressing INs (Hosp et al., 2014). We investigated the impact of PKA on GC GABA synapses originated in PV INs (PV_GC_) and SST INs (SST_GC_). We used optogenetics to evoke GABA release in acutely prepared slices from PV-Cre and SST-Cre transgenic mice previously infected with AAVs carrying a Cre-dependent modified channelrhodopsin (ChETA-EYFP). After establishing a recording of a GC, we used an LED to deliver a brief (2 ms) 490 nm light pulse to activate ChETA-expressing SST_GC_ and PV_GC_ inhibitory synapses (Fig. 2*A*).

We detected time-locked responses in GCs to light stimulation of SST_GC_ and PV_GC_ synapses, which we named optogenetically evoked IPSCs. Analysis of the kinetics showed that the rise slope of oeIPSCs was higher in PV_GC_ synapses compared with SST_GC_ synapses (Fig. 2*B*; PV_GC_, 0.35 ± 0.03; SST_GC_, 0.27 ± 0.02 pA/ms; two-sided *t* test, *t*_(78)_ = 2.11, *p* = 0.04; GCs from PV-Cre mice, *n* = 44; GCs from SST-Cre mice, *n* = 36). Reconstruction of the recorded GCs and visualization of the EYFP-tagged ChETA showed that the majority of the axonal arborization from PV INs accumulated around GC soma, in the GC layer. In contrast, axonal arborization from SST INs concentrated in the outer part of the ML, where GCs extend their dendrites (Fig. 2*C*). Thus, this optogenetic-based approach allows the selective stimulation of different sets of GABA synapses of GCs with different presynaptic origin, different axonal distribution, and different synaptic current kinetics.

We next tested the effect of PKA activation on SST_GC_ and PV_GC_ synapses. We observed that the amplitude of oeIPSCs of PV_GC_ synapses was significantly increased after transient application of the PKA activator FSK in only 1 of the 12 recorded GCs (Fig. 2*D*,*E*). At the population level, FSK failed to increase oeIPSC amplitude of PV_GC_ synapses compared with the baseline period (Bsl, 103 ± 2%; FSK, 90 ± 10%; *p *=* *0.73; Fig. 2*F*). In contrast, the oeIPSC amplitude of SST_GC_ synapses increased in 8 of 10 recorded GCs after transient FSK application (Fig. 2*D*,*E*). The amplitude of the oeIPSC of SST_GC_ synapses changed from 99 ± 2% during the baseline period to 192 ± 20% after FSK application (*p *<* *0.0001; Fig. 2*F*). FSK failed to significantly increase the amplitude of the oeIPSC of SST_GC_ synapses in slices treated with PKA inhibitor H89 (SST_GC_ + H89; Fig. 2*D–F*).

These results suggest that PKA-induced iLTP in GCs preferentially affects GABA synapses originated in SST INs, but not in those originated in PV INs.

### PKA-induced iLTP is accompanied by changes in PPR

**Figure 3.**
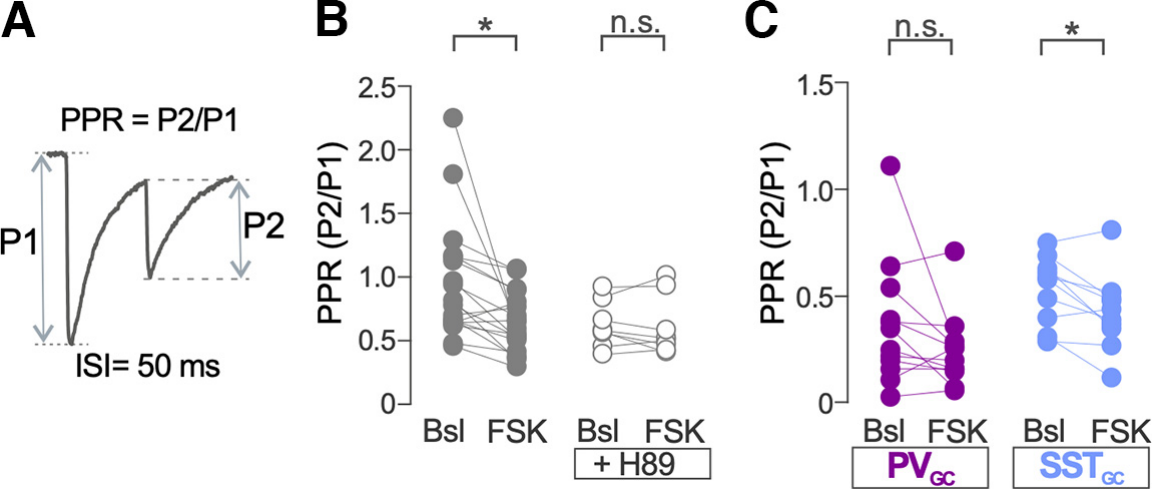
PKA-induced iLTP is accompanied by changes in PPR. ***A***, Schematic of the method used to calculate PPR for eIPSCs and oeIPSCs. ISI, Interstimulus interval. ***B***, Population data for eIPSCs PPR calculated before (Bsl) and after FSK application in the presence and absence of PKA inhibitor H89. Two-way ANOVA; FSK versus FSK+H89: *F*_(1,24)_ = 2.57, *p* = 0.12; Bonferroni’s multiple-comparisons test; Bsl versus FSK, *p* = 0.003; Bsl versus FSK+H89, *p* = 0.99; FSK, *n* = 18 GCs; FSK + H89, *n* = 8 GCs. ***C***, Population data for oeIPSC PPR calculated before (Bsl) and after FSK application in PV_GC_ and SST_GC_ synapses. Two-way ANOVA; PV_GC_ versus SST_GC_: *F*_(1,20)_ = 4.71, *p* = 0.04; Bonferroni’s multiple-comparisons test; PV_GC_ Bsl versus FSK, *p* = 0.29; SST_GC_ Bsl versus FSK, *p* = 0.03; PV-Cre mice, *n* = 12 GCs; SST-Cre mice, *n* = 10 GCs. *p < 0.05; n.s., nonsignificant. Dots in ***B*** and ***C*** represent individual measurements.

We next investigated changes in presynaptic function associated to PKA-dependent iLTP observed in GABAergic synapses in GCs. We calculated the PPR of the response amplitude (eIPSCs or oeIPSCs) to a pair of stimuli delivered 50 ms apart (Fig. 3*A*). Changes in the PPR are assumed to reflect changes in the probability of neurotransmitter release. The PPR of eIPSCs on GCs was significantly decreased after transient application of FSK (*p *=* *0.003; Fig. 3*B*). The FSK-induced change in PPR was not observed in the experiments performed in the presence of the PKA inhibitor H89 (*p *=* *0.99; Fig. 3*B*). We performed a similar analysis in oeIPSCs from GCs after optogenetic stimulation of SST_GC_ and PV_GC_ synapses. We observed that the baseline PPR of oeIPSCs from PV_GC_ synapses was significantly smaller with respect to the PPR of oeIPSCs from SST_GC_ synapses (mean ± SEM; PPR: PV_GC_, 0.34 ± 0.05; SST_GC_, 0.74 ± 0.09; Mann–Whitney test: *U *=* *48.5, *p *<* *0.0001; *n *=* *22 and *n* = 16, respectively). Transient application of FSK significantly reduced the PPR of oeIPSC from SST_GC_ synapses (*p *=* *0.03; Fig. 3*C*), but not the PPR of oeIPSCs from PV_GC_ synapses (*p *=* *0.29; Fig. 3*C*).

**Figure 4.**
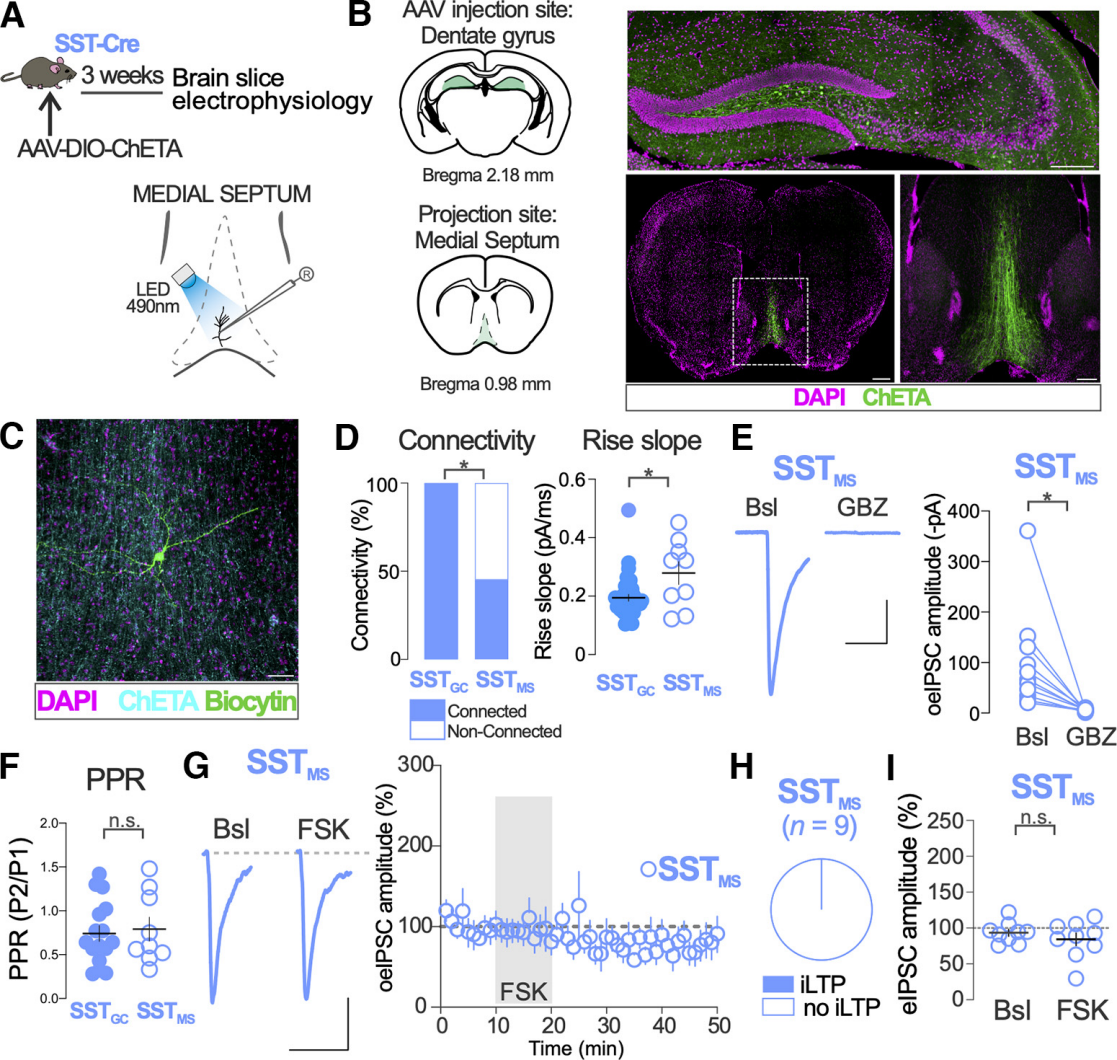
Synaptic properties and PKA-dependent plasticity of DG SST IN distal synapses on medial septal neurons (SST_MS_)_._ ***A***, Schematic of experimental procedure. ***B***, Representative images of AAV-dependent ChETA expression in SST-Cre mice injected in the DG. Top, The injection site of AAV in the DG. Scale bar, 200 μm. Bottom, Low-magnification (scale bar: left, 500 μm) and high-magnification (scale bar: right, 200 μm) of DG SST IN long-range projections in the MS. Anatomical diagrams approximately corresponding to the images shown are adapted from Paxinos and Franklin (2012). ***C***, Representative image of medial septum slice showing a biocytin-filled MS neuron (green). Long-range axonal projections from hilar ChETA expressing SST IN are shown in cyan. Scale bar, 50 μm. ***D***, Connectivity and rise slope comparison between distal SST_MS_ and local SST_GC_ synapses. Connectivity: two-sided Fisher’s exact test, *p* < 0,001; rise slope: two-sided Mann–Whitney test: *U* = 89.5, *p* = 0.04; SST_GC_, *n* = 36 synapses; SST_MS_, *n* = 9 synapses. ***E***, The GABA_A_ receptor blocker gabazine (GBZ) blocks oeIPSC responses in MS neurons to SST_MS_ synapses optogenetic stimulation. Two-sided Mann–Whitney test: *U* = 0, *p* < 0.0001. Calibration: 100 pA, 50 ms. ***F***, The PPR of SST_MS_ and SST_GC_ oeIPSCs did not differ. Two-sided Mann–Whitney test: *U* = 71, *p* = 0.97; SST_GC_, *n* = 16 synapses; SST_MS_, *n* = 9 synapses. ***G***, Representative traces of SST_MS_ oeIPSCs before (Bsl) and after FSK bath application. Calibration: 50 pA, 50 ms. The time course of FSK effect on normalized SST_MS_ oeIPSC amplitude is shown in the right graph. ***H***, Proportion of recorded MS neurons showing a significant increase of eIPSC amplitude after FSK application. ***I***, Population data of FSK effects on normalized SST_MS_ oeIPSC amplitude before (Bsl) and after FSK application. Two-sided *t* test: *t*_(16)_ = 0.93, *p* = 0.37; MS neurons, *n* = 9. *p < 0.05; n.s., nonsignificant. Dots in ***D***, ***E***, ***F***, and ***I*** represent individual measurements. Horizontal and vertical bars in ***D*** and ***I***, and circles and vertical bars in ***G*** represent the mean ± SEM.

**Figure 5.**
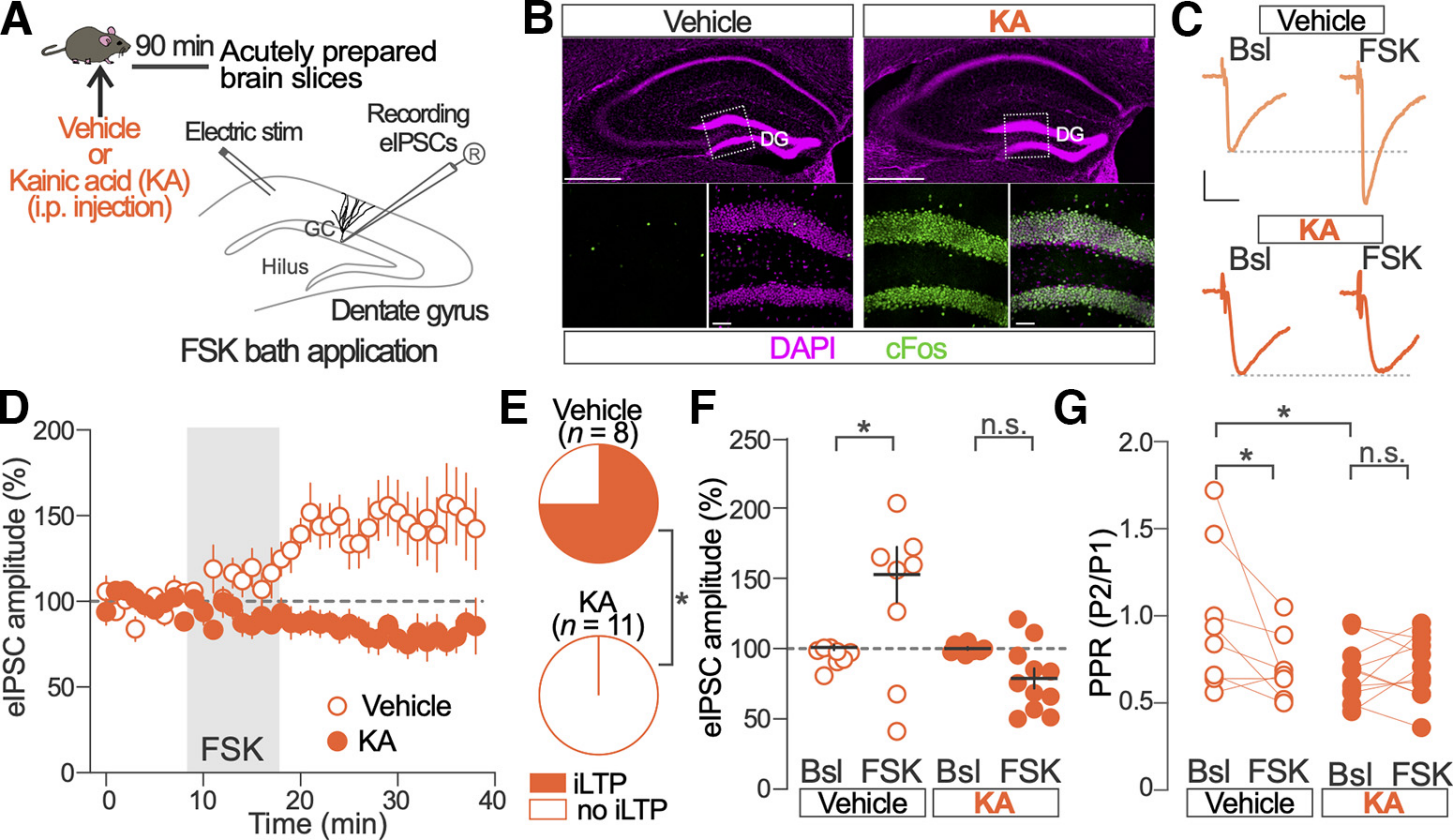
Kainic acid-induced increases in synaptic activity prevent the induction of iLTP in GCs. ***A***, Schematic of experimental procedure. ***B***, Representative example of c-Fos (green) expression in the DGs of vehicle- and KA-injected mice. Boxed regions in top panels are shown in bottom panels. Scale bars: top, 500 μm; bottom, 50 μm. ***C***, Representative traces of eIPSCs before (Bsl) and after FSK bath application recorded from GCs in vehicle- and KA-treated mice. Calibration: 25 ms, 50 pA. ***D***, Time course of FSK effects on normalized eIPSC amplitude in GCs from vehicle- and KA-treated mice. Vehicle-injected mice, *n* = 8 GCs; KA-injected mice, *n* = 11 GCs. ***E***, Proportion of recorded GCs showing a significant increase of eIPSC amplitude after FSK application in the vehicle- and KA-treated mice. Two-sided Fisher’s exact test, *p* = 0.001; vehicle-treated mice, *n* = 8 GCs; KA-injected mice, *n* = 11 GCs. ***F***, Population data of FSK effects on normalized eIPSC amplitude before (Bsl) and after FSK application in GCs from vehicle- and KA-injected mice. Two-way ANOVA; Vehicle versus KA: *F*_(1,17)_ = 15.85, *p* = 0.001; Bonferroni’s multiple-comparisons test; Vehicle Bsl versus FSK, *p* = 0.004; KA Bsl versus FSK, *p* = 0.19; vehicle-treated mice, *n* = 8 GCs; KA-injected mice, *n* = 11 GCs. ***G***, Population data for eIPSC PPR calculated before (Bsl) and after FSK application in GCs from vehicle- and KA-injected mice. Two-way ANOVA; Vehicle versus KA: *F*_(1,17)_ = 2.43, *p* = 0.14; Bonferroni’s multiple-comparisons test; vehicle Bsl versus FSK, *p* = 0.02; KA Bsl versus FSK, *p* = 0.99; Bsl Vehicle versus KA, *p* = 0.02; vehicle-treated mice, *n* = 8; KA-injected mice, *n* = 11 GCs. *p < 0.05; n.s., nonsignificant. Dots and vertical bars in ***D*** and horizontal and vertical bars in ***F*** represent the mean ± SEM. Dots in ***F*** and ***G*** represent individual measurements.

These data suggest that altered presynaptic release probability is involved in PKA-dependent iLTP of GABAergic synapses from SST INs.

### Synaptic properties and PKA-dependent plasticity of DG SST INs distal synapses on medial septal neurons (SST_MS_)

In addition to local axons that contact with GCs, DG SST INs extend collaterals through the fimbria that form synapses with MS neurons (Jinno, 2009). We sought to compare synaptic properties and PKA-dependent plasticity of long-range [DG SST INs distal synapses on MS neurons (SST_MS_)] and local (SST_GC_) synapses established by DG SST INs. We used bilateral injections of AAVs ChETA-EYFP in the DG of SST-Cre mice to anterogradely label and manipulate DG SST IN projections to the MS (Fig. 4*A*). Consistent with previous reports, fibers with abundant varicosities were observed in the MS of injected mice (Fig. 4*B*). We used acutely prepared slices and patch-clamp recordings of MS neurons coupled to optogenetics to determine the properties of and study PKA-dependent plasticity of SST_MS_ synapses, as we did previously with SST_GC_ synapses (Fig. 4*C*).

We first determined the connectivity and synaptic properties of SST_MS_ synapses. The proportion of recorded MS neurons that showed time-locked responses to SST_MS_ synapse activation was 45% (39 of 86 MS neurons tested). In contrast, the response to SST_GC_ synapse activation was observed in all GCs tested (36 of 36 GCs tested; Fig. 4*D*, Connectivity; *p *<* *0.0001). The rise slope of oeIPSCs from SST_MS_ synapses was significantly higher compared with SST_GC_ synapses (Fig. 4*D*, Rise Slope; *p *=* *0.04). The response to optogenetic activation of SST_MS_ synapses was completely abolished by the GABA_A_ receptor blocker gabazine (*p *<* *0.0001; Fig. 4*E*), suggesting that, similar to SST_GC_ synapses, responses to SST_MS_ synapses are mediated by the release of GABA. The PPR of oeIPSCs of SST_MS_ synapses was similar to that of SST_GC_ synapses (Fig. 4*F*; *p *=* *0.97), suggesting no differences in the presynaptic probability of release between these two groups of synapses.

We next tested the role of PKA in regulating the plasticity of SST_MS_ synapses. The amplitude of oeIPSC of SST_MS_ synapses was not significantly altered in any of the nine MS neurons tested after transient (10 min) application of FSK (Fig. 4*G*,*H*). At the population level, FSK failed to increase oeIPSC amplitude of SST_MS_ synapses in all MS neurons tested (Bsl, 93 ± 5%; FSK, 84 ± 9%; *p *=* *0.37; Fig. 4*I*). FSK did not alter the PPR of oeIPSCs of SST_MS_ synapses (Bsl, 0.79 ± 0.13%; FSK 0.65 ± 0.11%; *p *=* *0.29; *n *=* *9 MS neurons).

These results show connectivity and functional differences between SST_GC_ and SST_MS_ synapses and suggest that PKA-dependent iLTP affects local (SST_GC_) but not long-range (SST_MS_) synaptic contacts established by DG SST INs.

### Kainic acid-induced increases in neuronal activity prevents the induction of iLTP in GCs

In the hippocampus, neuronal activity induces multiple forms of GABAergic synapse plasticity (Castillo et al., 2011; Méndez and Bacci, 2011). For this reason, we tested the role of neuronal activity in regulating PKA-dependent iLTP of GC inhibitory synapses *in vivo*. We pharmacologically increased synaptic activity by injecting mice with KA (5 mg/kg; two to three injections; Fig. 5*A*). KA leads to the induction of the activity-regulated transcription factor c-Fos in virtually all GCs 90 min after the injection, in contrast with the sparse presence of c-Fos in GCs from vehicle-treated mice (Fig. 5*B*). These results indicate a widespread increase in neuronal activity in GCs after KA injection. We determined the effect of *in vivo* neuronal activity enhancement on FSK-induced iLTP and inhibitory presynaptic function. For this, we recorded eIPSCs in GCs from brain slices acutely prepared 90 min after vehicle or KA injections.

We first tested the induction of iLTP with FSK in GCs from vehicle- or KA-injected mice. Transient application of FSK failed to induce iLTP in slices from KA-treated mice, in contrast with slices from vehicle-injected control animals (Fig. 5*C*,*D*). FSK induced iLTP in 6 of 8 GCs recorded from vehicle-injected mice, while none of the 11 GCs recorded from KA-injected animals showed apparent increases in eIPSC amplitude (Fig. 5*E*). While the amplitude of eIPSC in vehicle-injected animals increased after FSK treatment (Bsl, 101 ± 2%; FSK, 153 ± 20%; *p *=* *0.004; Fig. 4*F*), the amplitude of eIPSCs from KA-injected animals was not significantly increased by FSK application (Bsl, 100 ± 1%; FSK, 79 ± 7%; *p *=* *0.18; Fig. 5*F*).

We then tested the PPR of pairs of eIPSCs delivered 50 ms apart in GCs from vehicle and KA-injected mice (Fig. 5*A*). We first compared the PPR of the eIPSCs in baseline conditions and found that eIPSC in slices from KA-injected animals had reduced PPR compared with vehicle-treated animals (*p *=* *0.02; Fig. 5*G*). The PPR of eIPSCs of KA-treated animals did not change in response to FSK treatment (*p *>* *0.99; Fig. 5*G*), while the PPR of eIPSCs in GCs from vehicle-treated mice decreased after FSK application (*p *=* *0.02; Fig. 5*G*).

These results show that increasing synaptic activity with KA treatment *in vivo* prevents FSK-induced iLTP. In addition, the reduced baseline PPR of eIPSCs in GCs from KA-injected mice suggests that *in vivo* increases in neuronal activity occlude the induction of iLTP in GCs.

## Discussion

The functional role of inhibitory synapse plasticity needs to be understood from the point of view of the heterogeneity of inhibitory cell types that establish GABAergic synapses. Our results reveal a novel plasticity mechanism of GC GABAergic synapses that may be linked to the identity and projection characteristics of presynaptic INs. Activation of PKA through cAMP production leads to the potentiation of a fraction of GABAergic synapses of GCs, those originated in local SST INs, but leaves GABAergic synapses from PV INs unaffected. In contrast to local GC synapses, long-range synapses formed by SST IN collaterals in MS are not affected by PKA activation. PKA-dependent iLTP reduces the PPR of SST INs GABAergic synapses in GCs, but it did not affect PPR of PV IN synapses onto GCs or the PPR of distal SST IN synapses onto MS neurons. This suggests that a change in the presynaptic release is part of the mechanism of PKA-dependent GABAergic synapse plasticity in GCs. Our experiments with systemic KA injections suggest that increasing hippocampal network activity *in vivo* occludes iLTP of GABAergic synapses on GCs. This is in line with a fundamental role of activity in triggering GABAergic synapse plasticity *in vivo* and point to a potential role of GABAergic plasticity in experience-dependent plasticity mechanisms.

cAMP regulates the probability of glutamate release of GC output synapses onto CA3 pyramidal cells (Monday et al., 2018). The control over GABAergic synapse plasticity that we describe here suggests that PKA may be a central mediator of the mechanisms that regulate excitatory and inhibitory synapse strength and may in this way contribute to adjust the excitatory/inhibitory balance in hippocampal circuits. Potentiation was observed in outward IPSCs recorded with low-chloride solution, suggesting than this form of plasticity is independent of the reversal potential for GABA_A_ receptor currents in mature GCs (Woodin et al., 2003) and argues against the requirement of postsynaptic GABAergic synaptic responses in this form of plasticity. In addition to the control of synaptic function, the activation of PKA induces the formation of GABAergic terminal boutons in the hippocampus (Liang et al., 2021), suggesting additional plasticity mechanisms involving structural remodeling of GABA synapses.

Several subcortical neuromodulatory systems targeting the hippocampus may activate, alone or in combination, receptors with potential to regulate cAMP synthesis and, thus, PKA activity, including dopamine, adrenergic, and cholinergic receptors (Greengard, 2001). Our results suggest a new mechanism by which neuromodulatory signaling from subcortical structures may regulate the plasticity of inhibitory synapses and hippocampal information processing. In addition, INs are exquisitely sensitive to different forms of neuronal activity that activate multiple signaling pathways resulting in changes in gene expression, synapse structure, and efficacy (Spiegel et al., 2014).

Different subsets of GABAergic synapses on GCs are endowed with specific plasticity mechanisms that are linked to the identity of the presynaptic INs. PV and SST INs are major contributors to synaptic inhibition in the DG (Hainmueller and Bartos, 2020). Despite their common developmental origin in the medial ganglionic eminence (Lim et al., 2018), the output synapses of these two types of INs are differently affected by plasticity-inducing stimuli in the DG (this study) and CA1 (Udakis et al., 2020). Along with postsynaptic calcium (Udakis et al., 2020), our results show that presynaptic mechanisms that change the probability of release participate in altering synaptic efficacy of plasticity of SST INs in GCs, similar to PKA-dependent plasticity of synapses in other brain regions (Melis et al., 2002; Nugent et al., 2009; Bocklisch et al., 2013). PKA is widely expressed in excitatory and inhibitory hippocampal neurons which makes it unlikely that the specificity of PKA-dependent plasticity of synapses originating in SST INs arises from selective expression of the catalytic or regulatory PKA subunits. It is conceivable, however, that differences in the expression and subcellular localization of PKA-anchoring proteins (AKAPs) may differ in PV and SST INs. Of particular interest is AKAP7, abundantly expressed in the hilus and molecular layer, coinciding with the location SST cell bodies and axonal arborizations, since this protein is essential for PKA-induced presynaptic potentiation of GC glutamatergic synapses (Jones et al., 2016). Selective expression or axonal targeting of AKAP7 in SST INs may explain the preferential expression of PKA-dependent iLTP in GABAergic synapses of GC originated in this IN population. In addition to synapses from SST INs, eIPSCS reflect the activity of synapses originated in several types of DG INs. Some of these synapses may be also FSK sensitive and increase amplitude with a faster time course compared with those originated in SST INs. This is compatible with the fact that eIPSC amplitude continues to increase 10–20 min after FSK washout, when oeIPSCs from SST INs (SST_GC_) have the steepest increase in amplitude.

The connectivity and functional difference between SST_GC_ and SST_MS_ synapses point to differences in DG SST IN regulation of local hippocampal and extrahippocampal activity. The projection to the MS has been proposed to arise from a subset of DG SST INs that are different from Hilar perforant path-associated SST INs that exclusively target hippocampal GCs (Yuan et al., 2017). Our results extend this dichotomy between locally and distally projecting DG SST INs by showing that connectivity, postsynaptic response kinetics, and plasticity mechanisms are also different between SST_GC_ and SST_MS_ synapses. Sparse connectivity of SST_MS_ synapses is probably the reflection of innervation specificity among MS neuronal types (Yuan et al., 2017). The rapid kinetics and high probability of release of SST_MS_ synapses suggest the relevance of these synapses in coordinating the timing of hippocampal activity paced by the MS, in particular for theta oscillations (Jinno, 2009). Our results suggest that plasticity mechanism can be of help to understand the functional diversity of SST INs, which is likely present in other hippocampal (CA1, CA3) and cortical brain regions (Pelkey et al., 2017; Hostetler et al., 2023).

Several reports have shown that hippocampal GABAergic synapses undergo activity-dependent plasticity. Our results suggest that the potentiation of GC synapses originated in local SST INs can be induced by pharmacological increases of neuronal activity *in vivo*. This observation suggests that physiological (i.e., behaviorally induced) activity may lead to changes in GC GABA synapses and refine the important role of hippocampal INs in different stages of experience-dependent information processing and updating (Topolnik and Tamboli, 2022) by specifically modifying connectivity with excitatory neurons. Although our experiments do not directly demonstrate the involvement of PKA in kainate-induced potentiation of GABAergic synapses, the specificity of PKA, an important regulator of memory processes, in regulating the activity of dendrite-targeting SST INs is in line with the important role of these subtypes of INs in regulating DG excitability and memory allocation and recall (Stefanelli et al., 2016; Cummings and Clem, 2020). The control over synaptic inhibition exerted by PKA-dependent iLTP could allow a limitation to disinhibitory mechanisms through strengthening feedback, feedforward, and lateral inhibition onto GCs. In addition, SST INs have a critical role in controlling c-Fos expression and in regulating plasticity of excitatory synapses and excitability of GCs. The control of GC function by synaptic inhibition regulated by PKA could have important consequences to control the fraction of active GCs and the excitatory synapse function required to adjust the tight balance between pattern separation and completion in hippocampal circuits.

In summary, our results show a form of activity-dependent inhibitory synapse plasticity that is selectively expressed in local, but not in long-range, targets of hilar SST INs and regulates inhibitory microcircuits essential for hippocampal function.

## Synthesis

Reviewing Editor: Isabel Perez-Otaño, Instituto de Neurociencias Decisions are customarily a result of the Reviewing Editor and the peer reviewers coming together and discussing their recommendations until a consensus is reached. When revisions are invited, a fact-based synthesis statement explaining their decision and outlining what is needed to prepare a revision will be listed below. The following reviewer(s) agreed to reveal their identity: Kenneth Pelkey.

Both reviewers foudn merit in your work, but suggested a number of experiments that we belive will improve the work.

Reviewer #1

In this study, the authors investigated inhibitory synaptic transmission in the dentate gyrus of adult mice. Using pharmacology, electrophysiology, and optogenetics in acute brain slices, they tested the role of PKA activation in modulating synaptic inhibition in dentate granule cells (GCs). Taking advantage of optogenetics in PV-cre and SST-cre mice, they focused on the role of PV and SST interneurons, respectively. The main findings can be summarized as follows. Transient application of the adenylyl cyclase activator forskolin (for 10 min) potentiated IPSCs electrically evoked by stimulating in the outer molecular layer. This chemical potentiation lasted for 20 minutes and was associated with decreased PPR, suggesting a presynaptic mechanism. Using optogenetics, the authors report that the forskolin-induced potentiation occurs at GABAergic synapses that originated in SST but not in PV interneurons. Forskolin application did not affect SSTMS GABAergic transmission either. Lastly, the forskolin-induced potentiation was occluded in kainic acid-injected animals undergoing seizures. The authors concluded that PKA causes a form of long-term potentiation of inhibitory transmission (iLTP) in an input-specific manner, and that this potentiation can be elicited in vivo by strong neuronal activity. Overall, the experiments are well-designed, the results are clean, and support most of the conclusions that are made. However, the authors should address a few points to strengthen their findings.

MAJOR POINTS:

1) The study heavily relies on one PKA activator only. They may want to confirm the key results using another selective PKA activator such as a cell-permeable Camp analog. Because H89 prevented forskolin-induced potentiation, the authors concluded that this potentiation is mediated by PKA. However, H89 is known to have other targets. The authors may also want to strengthen their conclusion by using the cell-permeable PKA inhibitor myristoylated PKI14-22.

2) What is the effect of H89 application alone? The summary plot in Fig 1B suggests that H89 could reduce inhibitory transmission. It is unclear why the authors measured inhibitory transmission for only 15 minutes after forskolin wash-out. PKA inhibition could induce a form of chemical iLTD.

3) The time-course of the forskolin-induced potentiation is remarkably different in optically-evoked (SST-cre mice) vs electrically-evoked IPSCs. What accounts for this difference? Also, the authors should test whether the potentiation of light-evoked IPSCs is blocked by a selective PKA inhibitor.

4) The one-way occlusion reported in fig 5 weakly suggests but does not prove that PKA-mediated iLTP may be triggered in vivo by experience. There is no evidence that PKA mediates the potentiation induced by kainic acid injection. The authors may want to tone down their conclusions (see line 72).

5) How do the authors explain the apparent difference in basal PPR in electrically-evoked vs light-evoked (SST GC) IPSCs (Fig 3B,C)? When was PPR measured post-forskolin application?

6) What are the functional consequences of the forskolin-induced potentiation of GABAergic transmission in the dentate gyrus circuit? The authors should discuss this point more thoroughly.

7) Were the experimenters blind to condition during data acquisition and analysis?

MINOR POINTS:

- Line 78: the age of the animals in methods section does not match the age reported in figure 1.

- Line 76: what was the ratio between males and females used in these experiments?

- What was the LED power range used? Was it similar in PV vs SST experiments?

- L229: remove “plasticity”

Reviewer#2:

In this study the authors provide evidence that somatostatin+ (SST) interneuron mediated GABAergic input to the distal dendrites of dentate granule cells (GCs) undergoes sustained presynaptic potentiation (iLTP) following a transient increase in cAMP/PKA activity (pharmacologically driven with forskolin). In contrast, perisomatic GABAergic input to GCs provided from parvalbumin+ (PV) interneurons is insensitive to forskolin. Moreover, SST interneuron long-range extrahippocampal projections to the medial septum are also insensitive to forskolin. Finally, evidence is provided that in vivo kainic acid treatment to drive neuronal activity prior to making in vitro slices for synaptic evaluation occludes forskolin sensitivity of SST-GC GABAergic transmission. The findings of the study are likely to be of interest to a variety of neuroscientists interested in interneurons, synaptic physiology, synaptic plasticity, hippocampal network function and epilepsy. However, addressing the following experimental technical and logical concerns would greatly improve the manuscript.

1. For the electrical stimulation experiments performed the authors were careful to perform recordings with both a high and low Cl intracellular solution and explicitly state use of kynurenic acid. However, for optogenetic experiments it appears that only high Cl solution was used. Further it is not specifically indicated whether optogenetic experiments were performed with glutamatergic transmission blocked (kynurenate/APV etc). Because the SST-cre driver line is well recognized to promote recombination in significant numbers of pyramidal cells it is crucial that the authors take care to ensure that the optogenetically evaluated events using the SST-Cre driver line are free of glutamatergic contamination (either confirm full iGluR antagonism or work with a reversed polarity Cl-mediated event with “low Cl intracellular solution”).

2. The time course of forskolin induced SST interneuron iLTP for electrically stimulated events and optogenetically driven events is curiously divergent. Indeed, the ∼10min delay following termination of the forskolin application to observe an increase in the optogenetically driven SST-mediated IPSC is rather unusual. Forskolin typically promotes a strong potentiation of cAMP/PKA sensitive synapses within minutes during application that wanes during washout (for example see a very rich hippocampal mossy fiber literature).

3. Does H-89 application following forskolin induced potentiation reverse iLTP? Currently the authors have only examined concomitant H-89 with forskolin but testing them sequentially would inform as to whether continued persistent PKA activity is necessary following the increased cAMP trigger. In addition, it would be good to know if there is any basal PKA tone modulating SST output (ie. does H-89 on its own depress transmission?). Finally, is synaptic activation during the forskolin treatment required for iLTP induction?

4. As the authors note, it is likely that the hippocampal SST interneurons giving rise to extrahippocampal long range projections are distinct cells from those that innervate the distal dendrites of GCs (likely HIPP interneurons). Thus, their findings clearly illustrate that not all SST interneurons exhibit cAMP/PKA sensitivity. However, to determine if the currently discovered plasticity is a universal feature of similar SST interneurons it would be more logical to evaluate cAMP/PKA sensitivity of intrahippocampal distal dendrite targeting SST cohorts in CA3-CA1 (ie. OLM interneurons) or their neocortical counterparts (Martinotti cells).
